# Synchronous and Metachronous Multiple Gastric Epithelial Neoplasms Detected During Index Evaluation and Post‐Endoscopic Submucosal Dissection Surveillance: A Descriptive Comparison

**DOI:** 10.1002/deo2.70437

**Published:** 2026-09-23

**Authors:** Tomokazu Yoshio, Shuhei Fukunaga, Tomoyuki Nakane, Shinpei Minami, Tomonori Cho, Kozo Tsuruta, Hiroshi Tanaka, Ryousuke Gotou, Yusei Watanabe, Michita Mukasa, Miwa Sakai, Takumi Kawaguchi, Hidetoshi Takedatsu

**Affiliations:** ^1^ Department of Medicine Division of Gastroenterology, Kurume University School of Medicine Kurume Japan

**Keywords:** endoscopic submucosal dissection, gastric neoplasms, *Helicobacter pylori*, metachronous tumor, synchronous tumor

## Abstract

**Objectives:**

We compared synchronous and metachronous gastric tumors detected during index evaluation and surveillance after endoscopic submucosal dissection (ESD), focusing on Kimura‐Takemoto O‐3 atrophy. *Helicobacter pylori* eradication history and the neutrophil‐to‐lymphocyte ratio (NLR) were exploratory.

**Methods:**

We retrospectively reviewed consecutive patients who underwent ESD. Patients whose first additional lesion was detected simultaneously or within 1 year after index ESD were classified as having synchronous gastric tumors (SGT); those with no synchronous lesion whose first additional lesion was detected later were classified as having metachronous gastric tumors (MGT). Logistic regression estimated associations with SGT rather than MGT presentation.

**Results:**

Among 1064 screened patients, 256 had multiple lesions (SGT, *n* = 169; MGT, *n* = 87). SGT patients were older (median, 76 vs. 72 years; *p* = 0.005), and O‐3 atrophy was more frequent (34.3% vs. 20.5%; *p* = 0.045). In exploratory multivariable analysis, age (adjusted odds ratio [aOR], 1.67 per 10 years; 95% confidence interval [CI], 1.12–2.51) and O‐3 atrophy (aOR, 2.17; 95% CI, 1.01–4.65) were associated with SGT presentation. Eradication timing was unavailable, and NLR was not independently associated with SGT.

**Conclusions:**

O‐3 atrophy was more frequent when the first additional lesion was detected synchronously. SGT and MGT may represent timing‐defined phenotypes within a continuous atrophic field rather than biologically distinct entities. Extensive atrophy supports meticulous whole‐stomach inspection at index evaluation and sustained surveillance.

## 1 Introduction

Endoscopic submucosal dissection (ESD) is an established curative treatment for selected early gastric cancer and gastric epithelial neoplasms with negligible risk of lymph‐node metastasis [1, 2]. Because the background gastric mucosa remains after ESD, additional lesions may subsequently be detected during surveillance [3].

Additional lesions are commonly classified by detection timing. Lesions detected simultaneously with the index lesion or within 1 year after ESD are generally considered synchronous, whereas those detected later are considered metachronous [4]. Lim et al. evaluated age, sex, *Helicobacter pylori* status, tumor size and location, mucosal atrophy, and intestinal metaplasia in relation to multiple, synchronous, or metachronous neoplasia; small primary tumor size was associated with synchronous neoplasia, whereas absence of *H. pylori* infection was associated with metachronous neoplasia [5]. Lee et al. focused on incidence and clinicopathological concordance between index and additional tumors, reporting 44 synchronous and 100 metachronous cases and frequent concordance in longitudinal location, macroscopic type, and histology [6]. However, neither study directly examined whether the most extensive Kimura‐Takemoto grade, O‐3, differed between mutually exclusive groups defined by the first additional lesion.

Background mucosal changes, particularly severe atrophic gastritis related to longstanding *H. pylori* infection, may contribute to multiple gastric neoplasms [7, 8]. We therefore focused the principal novelty on a direct descriptive comparison of O‐3 atrophy between timing‐defined groups. Documented *H. pylori* eradication history and neutrophil‐to‐lymphocyte ratio (NLR) were included only as exploratory variables because exact eradication timing was unavailable and NLR is a nonspecific systemic marker [9, 10].

We descriptively compared synchronous gastric tumors (SGT) and metachronous gastric tumors (MGT) detected during index evaluation and post‐ESD surveillance. The primary objective was to characterize differences in O‐3 atrophy and other clinicopathological features between patients classified by the timing of their first additional lesion, without assuming distinct biological mechanisms or estimating future lesion incidence.

## 2 Methods

### 2.1 Study Design and Patients

**FIGURE 1:**
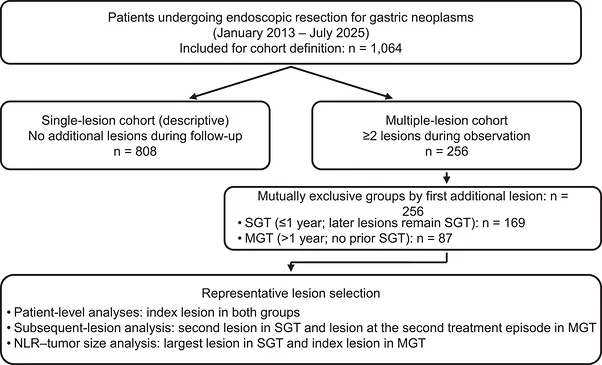
Flow diagram of patient selection, mutually exclusive group assignment based on the first additional lesion, and representative‐lesion selection. Patients assigned to synchronous gastric tumors (SGT) remained in that group even if a later lesion was detected more than 1 year after index endoscopic submucosal dissection (ESD).

This retrospective observational study was conducted at a single tertiary referral center and reviewed consecutive patients who underwent ESD for gastric tumors between January 2013 and July 2025. Only patients with at least 1 year of endoscopic follow‐up after index resection were included to permit classification of synchronous and metachronous lesions. Patients subsequently diagnosed with additional gastric tumors during follow‐up were identified for the principal analysis. The study flow, group definitions, and representative‐lesion selection are summarized in Figure 1. The Institutional Review Board of Kurume University Hospital approved the protocol (no. 25266) and waived written informed consent. The study was conducted in accordance with the Declaration of Helsinki.

### 2.2 Definitions and Mutually Exclusive Group Assignment

Gastric tumors comprised gastric adenoma and early gastric cancer. SGT was defined as an additional tumor detected simultaneously with the index lesion or within 1 year after initial ESD. MGT was defined as a newly detected tumor more than 1 year after initial ESD in a patient without a synchronous lesion. Group assignment was based on the first additional lesion. Patients with any additional lesion detected simultaneously or within 1 year were assigned to SGT and remained in that group even if a later lesion developed. The MGT group therefore comprised only patients with no synchronous lesion whose first additional lesion was detected more than 1 year after initial ESD. The two groups were thus mutually exclusive and represent the timing of first additional‐lesion detection rather than lifetime absence or presence of later metachronous lesions. The 1‐year cutoff has been used to distinguish lesions more likely to have been unrecognized at index endoscopy from those detected later in the residual mucosa [4]. Analyses were performed at the patient or lesion level according to purpose. Patient‐level comparisons used the index lesion in both groups. Additional‐lesion comparisons used the second lesion in SGT and the lesion treated at the second treatment episode in MGT. If multiple synchronous lesions were identified, the second lesion was selected for uniform comparison. For the exploratory correlation between NLR and tumor size, the largest SGT lesion was selected to reflect synchronous tumor burden, whereas the index MGT lesion was used. Figure 1 summarizes these definitions and purpose‐specific selections.

### 2.3 Endoscopic Procedures and Surveillance

Experienced endoscopists performed standard ESD. Surveillance endoscopy was generally conducted at least annually under the institutional protocol; all included patients therefore had at least 1 year of follow‐up. The entire stomach was examined using high‐resolution endoscopy, with image‐enhanced endoscopy at the endoscopist's discretion. Suspicious lesions underwent targeted biopsy, and gastric tumors were histologically confirmed.

### 2.4 Data Collection

Clinical, laboratory, and endoscopic data were extracted from medical records. Variables included age, sex, body mass index, comorbidities, medications, NLR, smoking, alcohol consumption, documented *H. pylori* eradication history, and background mucosal findings. Eradication history was defined by any available documentation during the study period. Because the exact date relative to index ESD could not be reliably confirmed, documented eradication may have occurred before or after index treatment and was not treated as a baseline exposure; insufficient records were classified as unknown. Atrophy at index treatment was graded using the Kimura‐Takemoto classification (C‐1 to O‐3). Lesion variables included detection timing, maximum diameter, macroscopic type and location according to the Japanese classification of gastric carcinoma, histology based on biopsy or resection specimens, and pathological depth when available.

### 2.5 Statistical Analysis

Continuous variables are reported as median (interquartile range) and categorical variables as number (percentage). Continuous variables were compared using the Mann‐Whitney U test (two groups) or Kruskal‐Wallis test (three groups); categorical variables were compared using the chi‐square or Fisher's exact test, as appropriate. The comparison among single‐lesion, SGT, and MGT groups was descriptive. Among patients with multiple lesions, the primary outcome variable was the binary presentation category, coded as SGT = 1 and MGT = 0. Principal effect measures were unadjusted and adjusted odds ratios (ORs) with 95% confidence intervals for SGT group membership. These measures describe associations with a timing‐defined category and do not estimate lesion incidence or causal risk.

Exploratory logistic regression described variables associated with SGT rather than MGT group membership. Because the multiple‐lesion sample was limited, variables for the reduced multivariable model were selected based on clinical relevance and model parsimony: age, sex, documented *H. pylori* eradication history, and severe endoscopic atrophy (O‐3 vs non‐O‐3). Eradication estimates were exploratory because timing relative to index ESD was unavailable. NLR was also exploratory, with an additional sensitivity multivariable model including NLR.

Regression analyses used complete cases, and model‐specific sample sizes are reported in the tables. Eradication history was modeled as yes, no, or unknown to avoid excluding patients solely because of incomplete records; a sensitivity analysis excluded unknown status. This addressed missing classification but not whether eradication occurred before or after index ESD. Atrophy was dichotomized as O‐3 versus non‐O‐3 to represent advanced open‐type atrophy and simplify the model.

Spearman rank correlation assessed NLR and lesion size, and Fisher's z transformation compared correlation coefficients between groups. The largest lesion was used in SGT and the index lesion in MGT. All tests were two‐sided, with *p* < 0.05 considered statistically significant. Analyses used JMP version 18 (SAS Institute Inc., Cary, NC, USA).

## 3 Results

### 3.1 Three‐Group Comparison: Single‐Lesion Versus Synchronous Versus Metachronous

Between January 2013 and July 2025, 1064 patients who underwent ESD for gastric neoplasms were screened. All included patients had at least 1 year of endoscopic follow‐up, and surveillance was generally annual. During follow‐up, 256 patients had multiple lesions: 169 were assigned to SGT and 87 to MGT according to the first additional lesion. Patients assigned to SGT remained in that group if a later lesion developed. The remaining 808 patients were summarized as the single‐lesion group (Table 1). The single‐lesion and MGT groups showed broadly similar distributions of baseline and index‐lesion characteristics, whereas the SGT group had a more distinct profile.

**TABLE 1:** Baseline characteristics and index lesion features across single‐lesion, synchronous, and metachronous groups.

	Single *N* = 808	Synchronous *N* = 169	Metachronous *N* = 87	*p*‐value
Age, years	73.0 [67.0, 79.0]	76.0 [69.0, 82.0]	72.0 [67.0, 78.0]	<0.001
Sex				
Male	569/808 (70.4%)	120/169 (71.0%)	64/87 (73.6%)	0.827
Female	239/808 (29.6%)	49/169 (29.0%)	23/87 (26.4%)	
Tumor size, long diameter (mm)	15.0 [10.0, 22.0]	20.0 [13.0, 27.0]	15.0 [11.5, 21.0]	<0.001
Tumor size, short diameter (mm)	10.0 [8.0, 16.0]	14.0 [10.0, 20.0]	11.0 [7.5, 15.0]	<0.001
Location				0.942
L	403/792 (50.9%)	86/165 (52.1%)	41/85 (48.2%)	
M	263/792 (33.2%)	55/165 (33.3%)	31/85 (36.5%)	
U	122/792 (15.4%)	24/165 (14.5%)	13/85 (15.3%)	
Other	4/792 (0.5%)	0/165 (0.0%)	0/85 (0.0%)	
Macroscopic type				0.012
IIc	405/796 (50.9%)	57/157 (36.3%)	39/86 (45.3%)	
IIa	160/796 (20.1%)	48/157 (30.6%)	24/86 (27.9%)	
IIa+IIc	123/796 (15.5%)	21/157 (13.4%)	10/86 (11.6%)	
IIc+IIa	42/796 (5.3%)	7/157 (4.5%)	6/86 (7.0%)	
I	23/796 (2.9%)	12/157 (7.6%)	4/86 (4.7%)	
IIb	21/796 (2.6%)	6/157 (3.8%)	1/86 (1.2%)	
Other	22/796 (2.8%)	6/157 (3.8%)	2/86 (2.3%)	
Histology				0.041
Differentiated	609/808 (75.4%)	118/161 (73.3%)	69/85 (81.2%)	
Adenoma	44/808 (5.4%)	13/161 (8.1%)	4/85 (4.7%)	
Mixed	95/808 (11.8%)	23/161 (14.3%)	9/85 (10.6%)	
Undifferentiated	48/808 (5.9%)	1/161 (0.6%)	2/85 (2.4%)	
Other	12/808 (1.5%)	6/161 (3.7%)	1/85 (1.2%)	
Depth of invasion				0.435
T1a/M	725/757 (95.8%)	127/136 (93.4%)	80/81 (98.8%)	
T1b/SM	22/757 (2.9%)	6/136 (4.4%)	1/81 (1.2%)	
Other/Unknown	10/757 (1.3%)	3/136 (2.2%)	0/81 (0.0%)	

An available case analysis was used.

Median age was 76 years (interquartile range [IQR], 69–82) in SGT, 73 years (67–79) in the single‐lesion group, and 72 years (67‐78) in MGT (*p* < 0.001). Index lesions were larger in SGT (long diameter, 20 mm [13–27]; short diameter, 14 mm [10–20]) than in the single‐lesion group (15 mm [10–22] and 10 mm [8–16]) or MGT (15 mm [11.5–21] and 11 mm [7.5–15]; both *p* < 0.001). Macroscopic type differed (*p* = 0.012), with type IIc less frequent in SGT (36.3% vs. 50.9%) and types IIa and I more frequent than in the single‐lesion group. Histology also differed (*p* = 0.041), with differentiated‐type histology most frequent in MGT. Sex, tumor location, and depth did not differ.

### 3.2 Comparison of Synchronous versus Metachronous Lesions in Multiple‐Lesion Patients

Patients with SGT were older than those with MGT (median, 76 years [IQR, 69–82] vs. 72 years [67–78]; *p* = 0.005), whereas sex, body mass index, comorbidities, medications, alcohol consumption, and smoking were similar (Table 2). The observation period was shorter in SGT (median, 3.30 years [2.28–5.00] vs. 6.05 years [3.78–8.11]; *p* < 0.001). Documented eradication history differed (*p* < 0.001): a record of eradication was more frequent in MGT (71.1% vs. 29.4%), whereas unknown status was more frequent in SGT (44.4% vs. 19.7%). Because timing relative to index ESD was unavailable, this finding was exploratory. The Kimura‐Takemoto distribution differed between groups (*p* = 0.045); O‐3 atrophy was more frequent in SGT (34.3% vs. 20.5%), whereas O‐2 was more frequent in MGT (44.6% vs. 30.7%). NLR was also higher in SGT (2.5 vs. 1.9; *p* = 0.012) but was exploratory.

**TABLE 2:** Baseline and laboratory characteristics of patients with synchronous versus metachronous gastric tumors.

	Synchronous *N* = 169	Metachronous *N* = 87	*p*‐value
Age, years	76.0 [69.0, 82.0]	72.0 [67.0, 78.0]	0.005
Sex			0.769
Male	120/169 (71.0%)	64/87 (73.6%)	
Female	49/169 (29.0%)	23/87 (26.4%)	
BMI, kg/m^2^	22.5 [20.5, 25.4]	23.3 [20.4, 25.8]	0.477
Hypertension	70/151 (46.4%)	40/87 (46.0%)	1
Diabetes mellitus	36/151 (23.8%)	20/87 (23.0%)	1
Dyslipidemia	33/151 (21.9%)	24/87 (27.6%)	0.346
Antithrombotic therapy	27/151 (17.9%)	12/87 (13.8%)	0.47
PPI/PCAB use	36/151 (23.8%)	22/87 (25.3%)	0.876
NSAID use	1/151 (0.7%)	2/87 (2.3%)	0.556
Endoscopic observation period, years	3.30 [2.28–5.00]	6.05 [3.78–8.11]	<0.001
H. pylori eradication			<0.001
Yes	45/153 (29.4%)	54/76 (71.1%)	
No	40/153 (26.1%)	7/76 (9.2%)	
Unknown	68/153 (44.4%)	15/76 (19.7%)	
Kimura–Takemoto classification			0.045
C‐1	3/140 (2.1%)	0/83 (0.0%)	
C‐2	11/140 (7.9%)	11/83 (13.3%)	
C‐3	13/140 (9.3%)	10/83 (12.0%)	
O‐1	22/140 (15.7%)	8/83 (9.6%)	
O‐2	43/140 (30.7%)	37/83 (44.6%)	
O‐3	48/140 (34.3%)	17/83 (20.5%)	
WBC, /µL	5400 [4600, 6800]	5500 [4400, 6750]	0.556
Albumin, g/dL	4.0 [3.8, 4.2]	4.0 [3.9, 4.3]	0.158
Creatinine, mg/dL	0.8 [0.7, 1.0]	0.8 [0.7, 0.9]	0.946
HbA1c, %	6.0 [5.7, 6.3]	6.0 [5.6, 6.4]	0.984
NLR	2.5 [1.7, 3.5]	1.9 [1.5, 2.7]	0.012
Alcohol drinking, *n*/*N* (%)	78/149 (52.3%)	46/87 (52.9%)	1.000
Alcohol consumption, g/day	10.0 [0–28.0]	14.0 [0–36.0]	0.919
Smoking history, *n*/*N* (%)	93/150 (62.0%)	52/87 (59.8%)	0.783
Smoking amount, cigarettes/day	10.0 [0‐20.0]	15.0 [0‐20.0]	0.634

An available case analysis was used. For H. pylori eradication history, the exact timing relative to index ESD was unavailable; comparisons are exploratory and do not represent baseline treatment effects.

### 3.3 Characteristics of Subsequent Lesions

Subsequent lesions were defined as the second lesion in SGT and the lesion treated at the second treatment episode in MGT (Table 3). Long diameter tended to be greater in MGT than SGT (12.0 vs. 10.5 mm; *p* = 0.071). Histology differed (*p* = 0.002): differentiated‐type histology was more frequent in MGT (82.8% vs. 67.6%), whereas adenoma was less frequent (3.4% vs. 17.3%). Location and depth were similar.

**TABLE 3:** Comparison of subsequent lesions: Synchronous (second lesion) versus metachronous (lesions detected at the second treatment episode in MGT).

	Synchronous *N* = 169	Metachronous *N* = 87	*p*‐value
Tumor size, long diameter (mm)	10.5 [8.0, 15.0]	12.0 [9.5, 16.0]	0.071
Tumor size, short diameter (mm)	8.0 [6.0, 12.0]	9.0 [7.0, 15.0]	0.099
Location			0.478
L	83/142 (58.5%)	46/86 (53.5%)	
M	34/142 (23.9%)	25/86 (29.1%)	
U	25/142 (17.6%)	14/86 (16.3%)	
Other	0/142 (0.0%)	1/86 (1.2%)	
Macroscopic type			0.308
IIc	51/129 (39.5%)	42/87 (48.3%)	
IIa	48/129 (37.2%)	28/87 (32.2%)	
IIa+IIc	6/129 (4.7%)	10/87 (11.5%)	
IIc+IIa	5/129 (3.9%)	3/87 (3.4%)	
IIb	6/129 (4.7%)	2/87 (2.3%)	
Other	4/129 (3.1%)	1/87 (1.1%)	
Histology			0.002
Differentiated	94/139 (67.6%)	72/87 (82.8%)	
Adenoma	24/139 (17.3%)	3/87 (3.4%)	
Mixed	10/139 (7.2%)	8/87 (9.2%)	
Undifferentiated	3/139 (2.2%)	4/87 (4.6%)	
Other	8/139 (5.8%)	0/87 (0.0%)	
Depth of invasion			0.367
T1a/M	101/108 (93.5%)	82/84 (97.6%)	
T1b/SM	2/108 (1.9%)	1/84 (1.2%)	
Other/Unknown	5/108 (4.6%)	1/84 (1.2%)	

An available case analysis was used.

### 3.4 Exploratory Analysis of Synchronous Versus Metachronous Presentation

In univariable analyses, age (OR, 1.46 per 10‐year increase; *p* = 0.014), NLR (OR, 1.25 per unit; *p* = 0.015), and O‐3 atrophy (OR, 2.07; *p* = 0.025) were associated with SGT presentation (Table 4A). Documented eradication history was associated with lower odds of SGT (OR, 0.15; *p* < 0.001), but this was not a baseline treatment effect. In the reduced multivariable model, age (aOR, 1.67 per 10 years; 95% confidence interval [CI], 1.12–2.51; *p* = 0.013) and O‐3 atrophy (aOR, 2.17; 95% CI, 1.01–4.65; *p* = 0.047) remained associated with SGT (Table 4B). Documented eradication history was inversely associated with SGT (aOR, 0.14; 95% CI, 0.05–0.38; *p* < 0.001), but this estimate cannot be interpreted as a baseline treatment effect. Sex was not associated with presentation. These ORs describe group membership and do not estimate future lesion incidence or causal risk.

**TABLE 4A:** Exploratory univariable logistic regression for synchronous presentation (vs. metachronous presentation).

	*N*	OR (95% CI)	*p*‐value
Age (per 10‐year increase)	255	1.46 (1.08–1.96)	0.014
Male sex	256	0.88 (0.49–1.57)	0.667
*H. pylori* eradication history: Yes versus No	229	0.15 (0.06–0.36)	<0.001
*H. pylori* eradication history: Unknown versus No	229	0.79 (0.30–2.11)	0.643
O‐3 versus non–O–3 atrophy	227	2.07 (1.10–3.89)	0.025
NLR (per 1‐unit increase)	204	1.25 (1.04–1.49)	0.015

**TABLE 4B:** Exploratory multivariable logistic regression for synchronous presentation (vs. metachronous presentation).

	*N*	aOR (95% CI)	*p*‐value
Age (per 10‐year increase)	215	1.67 (1.12–2.51)	0.013
Male sex	215	1.44 (0.69–2.97)	0.330
*H. pylori* eradication history: Yes versus No	215	0.14 (0.05–0.38)	<0.001
*H. pylori* eradication history: Unknown versus No	215	0.78 (0.27–2.29)	0.653
O‐3 versus non–O–3 atrophy	215	2.17 (1.01–4.65)	0.047

Odds ratios estimate associations with SGT (coded 1) versus MGT (coded 0) group membership; they do not estimate lesion incidence or causal risk.

Variables for the reduced multivariable model were selected based on clinical relevance and model parsimony: age, sex, documented H. pylori eradication history, and O‐3 atrophy.

Complete‐case analysis was used; therefore, the number of patients differed across models. The exact timing of documented H. pylori eradication relative to index ESD was unavailable; eradication estimates are exploratory.

After excluding unknown eradication status, the aORs were 0.12 (95% CI, 0.05–0.31; *p* < 0.001) for documented eradication and 2.50 (95% CI, 0.96–6.49; *p* = 0.059) for O‐3 atrophy (Table S1). The latter showed a similar direction but became borderline, likely because of the reduced sample size. This sensitivity analysis addressed missing eradication classification but not the unresolved timing; the eradication estimate therefore remained exploratory.

### 3.5 Exploratory Association Between NLR and Tumor Burden

Because NLR differed between SGT and MGT in the patient‐level comparison, we further explored its association with tumor size (Table 5). NLR correlated weakly with representative‐lesion diameter in SGT (Spearman *ρ* = 0.218; *p* = 0.015) but not MGT (*ρ* = 0.040; *p* = 0.740). However, the correlation coefficients did not differ significantly by Fisher's z‐test (*p* = 0.231). In the sensitivity multivariable model, NLR was not independently associated with SGT (aOR, 1.14; 95% CI, 0.94–1.39; *p* = 0.190; Table S2). Thus, the NLR findings were hypothesis‐generating.

**TABLE 5:** Correlation between neutrophil‐to‐lymphocyte ratio (NLR) and tumor long diameter (Spearman).

Cohort	*n*	Spearman's *ρ*	*p*‐value
Synchronous (simultaneous or within 1 year), most extensive lesion	125	0.218	0.015
Metachronous (>1 year; no prior synchronous lesion), initial lesion	71	0.04	0.74
Difference in rho (sync—meta), Fisher's z approx	125 vs 71	0.178	0.231

## 4 Discussion

In this retrospective descriptive comparison of timing‐defined multiple‐lesion groups, O‐3 atrophy was more frequent in SGT than MGT, and exploratory logistic regression showed higher odds of SGT group membership with older age and O‐3 atrophy. Documented *H. pylori* eradication history and NLR differed in univariable comparisons but were treated as exploratory findings. The results characterize group differences and do not establish predictors of future tumor development.

The findings both confirm and extend earlier reports [5, 6]. Lim et al. evaluated age, sex, *H. pylori* status, tumor size and location, mucosal atrophy, and intestinal metaplasia in relation to multiple, synchronous, and metachronous neoplasia. Lee et al. described continued cumulative detection and frequent concordance in location, macroscopic type, and histology. In contrast, our study directly compared mutually exclusive groups based on the first additional lesion and specifically evaluated the full Kimura‐Takemoto distribution. O‐3 atrophy was more frequent in SGT (34.3% vs 20.5%) and was associated with SGT group membership in the reduced model. This adds a practical, graded endoscopic marker of the background mucosal field; however, it does not establish a mechanistic boundary between SGT and MGT. Severe endoscopic atrophy is linked to gastric carcinogenesis and multiple gastric neoplasms [7, 11, 12, 13, 14, 15, 16, 17].

SGT and MGT should therefore not be regarded as established biologically distinct conditions. The 1‐year cutoff separates the timing of detection, but lesions on either side of this boundary may arise from the same atrophic field, and some lesions detected within 1 year may have been present but unrecognized at index endoscopy. The higher O‐3 proportion in SGT underscores meticulous examination of the entire stomach at index evaluation. It does not justify attention only during the synchronous period: metachronous lesions continued to be detected during longer follow‐up, and the same high‐risk mucosal field supports sustained whole‐stomach surveillance after ESD. Our data do not determine different surveillance intervals or intensity for SGT and MGT.

The marked difference in documented eradication history was exploratory. We extracted any available documentation, but the date relative to the index ESD was unreliable; some patients may have undergone eradication after index treatment. The difference may therefore reflect post‐ESD management and longer follow‐up opportunity rather than a baseline predictor or treatment effect. Excluding unknown status addressed missing classification but not timing. Although eradication reduces metachronous gastric cancer risk, residual risk persists, particularly with advanced atrophy [8, 18, 19, 20]. NLR was likewise exploratory: its correlation with tumor size was weak, between‐group correlations did not differ, and it was not independently associated with SGT. Because NLR is affected by systemic inflammation, comorbidities, medications, and tumor burden, it should be considered hypothesis‐generating rather than a clinically useful discriminator [9, 10].

This study has several limitations. First, it was retrospective and single‐center, with possible residual confounding. Second, MGT patients had substantially longer observation (median, 6.05 vs. 3.30 years), providing more opportunity to detect late lesions. We could not adequately account for this follow‐up opportunity, and the study therefore represents a descriptive comparison of early‐ versus late‐detected multiple‐lesion phenotypes rather than a risk‐factor or incidence analysis. Third, patients with both synchronous and later metachronous lesions remained in SGT, precluding estimation of subsequent metachronous risk among patients with SGT. Fourth, some lesions detected within 1 year may have been missed at index endoscopy; inspection time, systematic image‐enhanced endoscopy, endoscopist experience, and equipment changes were not recorded. Fifth, eradication data were incomplete and temporally ambiguous; excluding unknown status did not establish whether eradication occurred before or after index ESD. Sixth, lifestyle variables were unavailable for the single‐lesion group, family history was not systematically recorded, and purpose‐specific representative‐lesion selection may not capture total tumor burden.

In conclusion, O‐3 atrophy was more frequent among patients whose first additional lesion was detected synchronously than among those whose first additional lesion was detected more than 1 year later. SGT and MGT are timing‐defined detection phenotypes that may reflect a continuous field‐carcinogenesis process rather than separate biological entities. Extensive atrophy supports both meticulous whole‐stomach inspection at index evaluation and sustained post‐ESD surveillance. Prospective longitudinal studies that account for follow‐up time are needed before differential surveillance strategies can be recommended.

## Author Contributions

Tomokazu Yoshio and Shuhei Fukunaga contributed to the study conception and design. Tomokazu Yoshio, Shuhei Fukunaga, Tomoyuki Nakane, Shinpei Minami, Tomonori Cho, Hiroshi Tanaka, Ryousuke Gotou, Yusei Watanabe, Michita Mukasa, and Miwa Sakai contributed to data collection. Tomokazu Yoshio and Shuhei Fukunaga performed the data analysis and interpretation. Tomokazu Yoshio drafted the manuscript. Shuhei Fukunaga, Kozo Tsuruta, Takumi Kawaguchi, and Hidetoshi Takedatsu critically revised the manuscript for important intellectual content. All authors read and approved the final manuscript.

## Funding

The authors have nothing to report.

## Ethics Statement

**Approval of the research protocol by an Institutional Reviewer Board**: This study was approved by the Institutional Review Board of Kurume University Hospital (approval number: 25266) and was conducted in accordance with the Declaration of Helsinki.

## Consent

The requirement for written informed consent was waived because of the retrospective nature of the study.

## Conflicts of Interest

The authors declare no conflicts of interest.

## Supporting information




**Supporting Table 1**: Exploratory sensitivity analysis excluding patients with unknown *H. pylori* eradication status.
**Supporting Table 2**: Exploratory sensitivity multivariable analysis including NLR for synchronous presentation (vs. metachronous presentation).

## Data Availability

The data that support the findings of this study are available from the corresponding author upon reasonable request.
