# Supplementary material for: Synchronous and Metachronous Multiple Gastric Epithelial Neoplasms Detected During Index Evaluation and Post‐Endoscopic Submucosal Dissection Surveillance: A Descriptive Comparison
Source: DEN Open. 2026 Sep 23;7(1):e70437. doi: 10.1002/deo2.70437 (PMC13598793; doi:10.1002/deo2.70437)
Supplement: Supplementary file 1 [file DEO2-7-e70437-s001.docx]

Supplementary Table 1. Exploratory sensitivity analysis excluding patients with unknown H. pylori eradication status

| Variable | n | aOR (95% CI) | P value |
| --- | --- | --- | --- |
| Age (per 10-year increase) | 132 | 1.18 (0.70-1.98) | 0.531 |
| Male sex | 132 | 1.16 (0.47-2.87) | 0.744 |
| H. pylori eradication history: Yes vs No | 132 | 0.12 (0.05-0.31) | <0.001 |
| O-3 vs. non-O-3 atrophy | 132 | 2.50 (0.96-6.49) | 0.059 |

**Supplementary Table 2. Exploratory sensitivity multivariable analysis including NLR for synchronous presentation (vs metachronous presentation)**

| Variable | n | aOR (95% CI) | P value |
| --- | --- | --- | --- |
| Age (per 10-year increase) | 177 | 1.39 (0.88-2.17) | 0.156 |
| Male sex | 177 | 2.13 (0.87-5.25) | 0.099 |
| H. pylori eradication history: Yes vs No | 177 | 0.09 (0.03-0.27) | <0.001 |
| H. pylori eradication history: Unknown vs No | 177 | 0.59 (0.18-1.90) | 0.376 |
| O-3 vs. non-O-3 atrophy | 177 | 4.62 (1.74-12.25) | 0.002 |
| NLR (per 1-unit increase) | 177 | 1.14 (0.94-1.39) | 0.190 |
